# End of Life: A New Life Course Stage for Older Adults and Their Families?

**DOI:** 10.1093/geroni/igaa057.2229

**Published:** 2020-12-16

**Authors:** Deborah Carr

**Affiliations:** Boston University, Boston, Massachusetts, United States

## Abstract

Over the past two centuries, death has transitioned from an unexpected and uncontrollable event to a protracted process that requires individuals and families to make difficult decisions regarding where and under what conditions one will die. This new life course stage, spanning the period from diagnosis to death, provides older adults and their families an opportunity to prepare for difficult medical decisions, yet also may be a time marked by suffering and conflict. In this paper, I provide an overview of the technological, demographic, and legal context of end-of-life in the 21st century, and its implications for the quality of life for dying patients and their families. I underscore that historical shifts have created a context in which the quality of one’s end-of-life experiences and autonomy are stratified by race and socioeconomic status, creating challenges for older adults and their loved ones. I highlight implications for research, policy, and practice.

